# Enhanced Achilles Tendon Gliding Through Ultrasound-Guided Manual Therapy Post-surgical Repair: A Case Report

**DOI:** 10.7759/cureus.68705

**Published:** 2024-09-05

**Authors:** Akihisa Watanabe, Takahiro Machida, Yuki Matsukubo

**Affiliations:** 1 Rehabilitation, Machida Orthopaedics, Kochi, JPN; 2 Orthopaedics, Machida Orthopaedics, Kochi, JPN

**Keywords:** achilles tendon injury, ankle joint, case report, dynamic ultrasound, manual therapy technique, range of motion exercise

## Abstract

Achilles tendon rupture is a common injury with established surgical treatments, but optimizing postoperative recovery remains challenging. Dynamic tendon gliding is necessary for normal ankle function, yet its role in recovery is not fully understood. This report highlights a novel approach using dynamic ultrasound imaging and ultrasound-guided manual therapy to improve Achilles tendon gliding post-surgery.

A 65-year-old man presented eight weeks after surgical repair of a left Achilles tendon rupture. Despite full weight-bearing ability and normal range of motion, the patient exhibited persistent dysfunction, such as an inability to perform single-leg stands and single-leg heel raises. Suspecting a dynamic issue with the Achilles tendon, dynamic ultrasound revealed significant adhesion between the Achilles tendon and Kager’s fat pad. To address this, ultrasound-guided manual therapy, involving specific mobilization of the tendon under ultrasound visualization, was initiated. The patient underwent 16 sessions over eight weeks, during which real-time ultrasound confirmed gradual improvement in tendon gliding. Post-treatment, the patient achieved marked functional recovery, demonstrated by the ability to perform single-leg heel raises and toe walking. His Achilles tendon Total Rupture Score improved from 47 to 75 points, with sustained benefits observed at the 26-week follow-up.

Ultrasound-guided manual therapy targeting tendon gliding dysfunction improved functional recovery in this patient. This approach underscores the importance of addressing tendon gliding in rehabilitation protocols to optimize outcomes. Further research is needed to validate these findings with a broader patient population.

## Introduction

Achilles tendon rupture is a common injury affecting the lower extremity [1,2], with both surgical and conservative treatments recognized as effective [3]. However, achieving optimal postoperative outcomes remains an area of ongoing research, with rehabilitation methods gaining in importance [4].

Achilles tendon-Kager’s fat pad (KFP) gliding is important for normal ankle joint function, yet the impact of gliding dysfunction post-surgery is not well understood [5,6]. While prior studies have focused on structural outcomes, there is a limited exploration of dynamic gliding dysfunction [7,8].

This case report presents a novel approach to managing a patient after Achilles tendon rupture by employing dynamic ultrasound imaging to identify and evaluate Achilles tendon adhesion, followed by ultrasound-guided manual therapy to enhance tendon gliding mechanics. The report details a case in which these interventions not only improved tendon gliding but also resulted in significant functional improvement of the ankle joint.

## Case presentation

**Figure 1:**
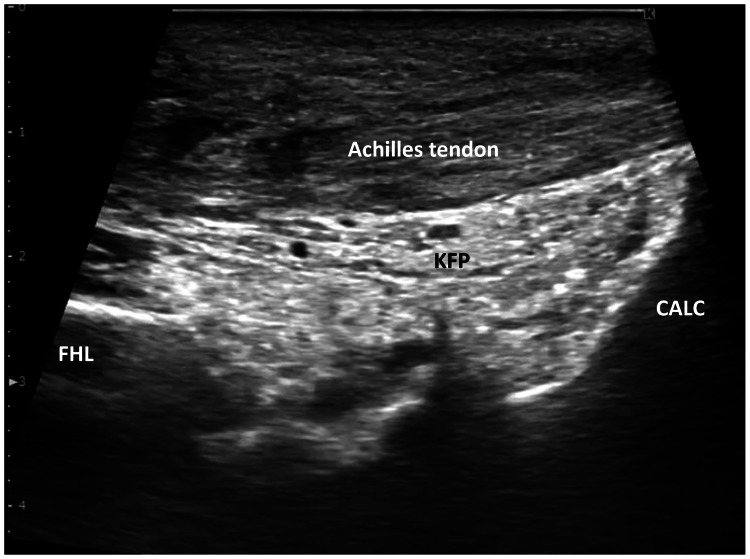
Static ultrasonography of the Achilles tendon before ultrasound-guided manual therapy (8-weeks post-surgery) CALC: calcaneus; FHL: flexor hallucis longus muscle; KFP: Kager’s fat pad.

A 65-year-old man presented eight weeks after surgical repair of a left Achilles tendon rupture using the Marti method [9]. Postoperative management included two weeks of immobilization in 5 degrees of plantarflexion, followed by initiation of weight-bearing and range-of-motion exercises. On initial evaluation, the patient demonstrated full weight-bearing ability without orthotics. The range of motion was 20 degrees in both passive and active dorsiflexion, 45 degrees in passive plantarflexion, and 20 degrees in active plantarflexion. The Thompson test was negative, and static ultrasonography confirmed the continuity of the Achilles tendon. Additional functional test results are provided in Table 1. However, dynamic ultrasonography during active plantarflexion revealed no gliding between the Achilles tendon and KFP, indicating adhesion (Figure 1, Video 1).

**Table 1:** Course of patient’s function ROM: range of motion; ATRS: Achilles tendon Total Rupture Score; 8wks to 26wks: post-surgery (weeks); sharp mark (#) indicates that the corresponding function test is possible, while blank cells indicate that the test was not possible.

	8wks	10wks	12wks	14wks	16wks	26wks
Active ROM, degrees						
Dorsiflexion	20	20	20	20	20	20
Plantarflexion	20	20	40	45	50	50
Passive ROM, degrees						
Dorsiflexion	20	20	20	20	20	20
Plantarflexion	45	45	45	45	50	50
ATRS	47	41	45	56	75	74
Function						
Both–leg heel raises	#	#	#	#	#	#
Single–leg heel raises			#	#	#	#
Single–leg standings		#	#	#	#	#
Toe walking					#	#
Jogging					#	#

**Video 1:** Dynamic ultrasonography in the Achilles tendon before ultrasound-guided manual therapy (8-week post-surgery) Gliding of the Achilles tendon and Kager’s fat pad is restricted.

To address this, ultrasound-guided manual therapy was applied to improve tendon gliding. The intervention, based on Cyriax’s cross-friction massage [10], utilized a 3-11 MHz B-mode linear array probe (SONIMAGE MX1, Konica Minolta, Tokyo, Japan) positioned along the tendon’s longitudinal axis. The therapist with over 10 years of experience in this technique manually facilitated gliding between the tendon and KFP while passively moving the ankle. Care was taken throughout the procedure to ensure that the patient experienced no pain. Each session lasted 20 minutes, and the patient was instructed to frequently perform active ankle movements as part of their home exercises. No additional modalities, such as hot packs, were used. The patient underwent 16 sessions over eight weeks.

**Figure 2:**
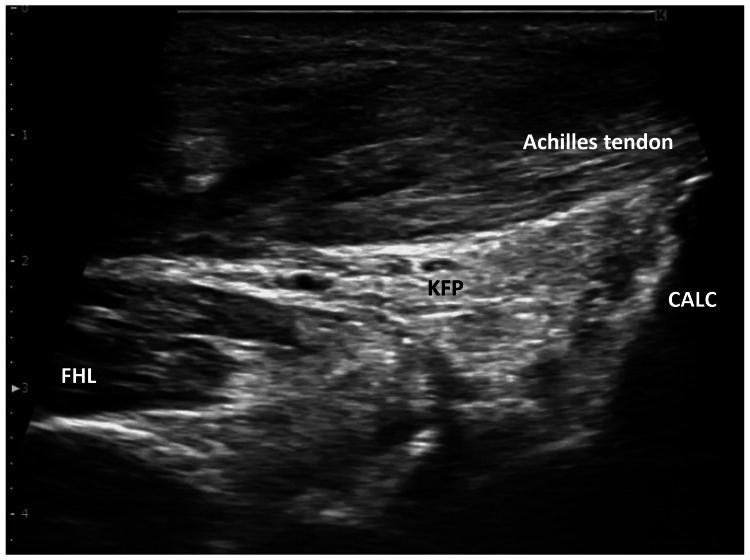
Static ultrasonography of the Achilles tendon after ultrasound-guided manual therapy (16-week post-surgery) CALC: calcaneus; FHL: flexor hallucis longus muscle; KFP: Kager’s fat pad.

Post-treatment assessments showed improved active plantarflexion. Functional improvements included the ability to perform single-leg heel raises and toe walking, with the Achilles tendon Total Rupture Score (ATRS) improving from 47 to 75 points (Table 1). Additionally, dynamic ultrasound evaluation showed improvement in gliding between the Achilles tendon and KFP during active plantarflexion (Figure 2, Video 2). At the 26-week follow-up post-surgery, the ATRS remained at 74 points, indicating sustained functional improvement.

**Video 2:** Dynamic ultrasonography in the Achilles tendon after ultrasound-guided manual therapy (16-week post-surgery) Gliding of the Achilles tendon and Kager’s fat pad is improved.

## Discussion

This case underscores the critical role of ultrasound-guided manual therapy in restoring Achilles tendon gliding, which may be pivotal for optimal postoperative recovery. Our intervention significantly improved both tendon gliding and overall function, as evidenced by the patient’s ATRS improvement. This highlights the importance of addressing tendon gliding dysfunction in postoperative management, which could lead to enhanced rehabilitation outcomes.

The literature focusing on tendon gliding in Achilles tendon rupture patients is limited. Previous studies have primarily emphasized restoring postoperative structural integrity and strength [11]. Some studies have explored tissue gliding in other conditions by using dynamic ultrasonography, such as addressing deep fascial adhesions that cause delayed onset muscle soreness [12] and improving scar tissue mobility after knee surgery by using ultrasound-guided hydrorelease [13]. Our findings support these studies, emphasizing the clinical relevance of focusing on tissue gliding to optimize patient outcomes.

Our ultrasound-guided manual therapy has several advantages: it does not require specialized therapeutic tools beyond the ultrasound imaging device, and both patients and therapists can monitor treatment progress by confirming the location of the gliding dysfunction and observing its improvement in real time.

The findings emphasize the critical need to address tendon gliding dysfunction in postoperative rehabilitation protocols to optimize functional recovery. Clinicians should consider dynamic assessments of gliding dysfunction as part of routine evaluation to improve long-term functionality in patients recovering from Achilles tendon rupture.

We acknowledge that the results of this case cannot be generalized to a broader population. While the KFP is known to play a shock-absorbing role in the ankle joint [6], our ultrasound imaging device was not capable of measuring the internal pressure or stiffness of the KFP area. Although KFP has been shown to have a proprioceptive role [6], the patient did not undergo proprioceptive testing. However, the restoration of heel raises and toe walking following ultrasound-guided manual therapy suggests that improving Achilles tendon gliding may also enhance proprioceptive sensation. Future studies measuring these effects will strengthen the validity of our ultrasound-guided manual therapy.

## Conclusions

In conclusion, the patient may achieve a successful outcome by focusing on tendon gliding dysfunction. Achieving optimal clinical outcomes in patients with Achilles tendon rupture requires not only structural assessment but also a comprehensive evaluation of tendon gliding mechanics, with targeted interventions integrated into rehabilitation protocols. Future research should focus on validating the effectiveness of ultrasound-guided manual therapies in diverse patient populations, with a particular emphasis on long-term functional outcomes and on the potential for integrating these techniques into standardized postoperative rehabilitation protocols.
